# Molecular Survey of *Babesia* and *Anaplasma* Infection in Cattle in Bolivia

**DOI:** 10.3390/vetsci8090188

**Published:** 2021-09-07

**Authors:** Shohei Ogata, Juan Antonio Cristian Pereira, Loza Vega Ariel Jhonny, Herbas Perez Gladys Carolina, Keita Matsuno, Yasuko Orba, Hirofumi Sawa, Fumihiko Kawamori, Nariaki Nonaka, Ryo Nakao

**Affiliations:** 1 Laboratory of Parasitology, Department of Disease Control, Faculty of Veterinary Medicine, Graduate School of Infectious Diseases, Hokkaido University, N 18 W 9, Kita-ku, Sapporo 060-0818, Japan; s.ogata@vetmed.hokudai.ac.jp (S.O.); nnonaka@vetmed.hokudai.ac.jp (N.N.); 2 Facultad de Ciencias Veterinarias, Universidad Autónoma Gabriel René Moreno, Av. 26 de Febrero Entre Av. Busch y Av. Centenario, Ciudad Universitaria, Modulo 228, Santa Cruz de la Sierra, Bolivia; antonios8@hotmail.com (J.A.C.P.); arlove@gmail.com (L.V.A.J.); carolinaherbas.ch@gmail.com (H.P.G.C.); iiiforest@zm.commufa.jp (F.K.); 3 Division of Risk Analysis and Management, International Institute for Zoonosis Control, Hokkaido University, N 20 W 10, Kita-ku, Sapporo 001-0020, Japan; matsuk@czc.hokudai.ac.jp; 4 International Collaboration Unit, International Institute for Zoonosis Control, Hokkaido University, N 20 W 10, Kita-ku, Sapporo 001-0020, Japan; orbay@czc.hokudai.ac.jp (Y.O.); h-sawa@czc.hokudai.ac.jp (H.S.); 5 One Health Research Center, Hokkaido University, N 18 W 9, Kita-ku, Sapporo 060-0818, Japan; 6 Division of Molecular Pathobiology, International Institute for Zoonosis Control, Hokkaido University, N 20 W 10, Kita-ku, Sapporo 001-0020, Japan

**Keywords:** *Anaplasma*, *Babesia*, Bolivia, cattle, ticks, tick-borne diseases

## Abstract

Latin American countries produce more than a quarter of the world’s beef and are a major global supplier of livestock protein. Tick-borne diseases (TBDs) are a major constraint to the livestock industry worldwide, including in Latin America. The aim of this study was to detect and characterise tick-borne pathogens in cattle from Santa Cruz, Bolivia, where no detailed epidemiological data are available. Blood samples were collected from 104 cattle. Apicomplexan parasites were detected by nested PCR amplification of the 18S ribosomal RNA gene (rDNA), and *Anaplasmataceae* was screened by the PCR amplification of 16S rDNA, followed by characterisation based on the heat shock protein and citrate synthase gene sequences. *Babesia* infection was observed in nine cattle (one *Babesia bovis* and eight *Babesia bigemina*), while *Anaplasmataceae* infection was detected in thirty-two cattle. A sequencing analysis confirmed the presence of *Anaplasma marginale* and *Anaplasma platys*-like. These results provide the first molecular evidence for the four above-mentioned tick-borne pathogens in cattle in Bolivia. This information improves our understanding of the epidemiology of TBDs and will help in formulating appropriate and improved pathogen control strategies.

## 1. Introduction

Latin American countries produce a substantial portion of the world’s beef supply [1]. Beef production in Latin America has increased by 29.8% over nearly two decades (between 2000 and 2018), and the livestock sector accounts for 46% of the agricultural gross domestic product in Latin America [1,2]. Diseases transmitted by ticks, so-called tick-borne diseases (TBDs), are a major issue in the livestock industry, causing considerable economic losses worldwide, including in Latin America. For instance, in Brazil, TBDs cause an annual economic loss of around 3.24 billion USD [3]. However, in many Latin American countries, the significance of TBDs has not been evaluated, in part owing to a lack of relevant epidemiological data.

In Latin American countries, the most severe and prevalent TBDs are babesiosis and anaplasmosis [4,5,6,7,8]. Bovine babesiosis is a globally distributed tick-borne hemoprotozoan disease caused by pathogenic species, such as *Babesia bovis*, *Babesia bigemina*, and *Babesia divergens*. The geographical distribution of the disease is defined by the prevalence of vector tick species [9]. The disease is clinically manifested by anaemia, fever, haemoglobinuria, and marked splenomegaly, sometimes resulting in death [10]. In Latin America, epidemiological studies have confirmed the presence of two pathogenic species, *B. bovis* and *B. bigemina*, which are transmitted by *Rhipicephalus microplus* [7]. The rate of tick transmission is generally higher for *B. bigemina* than for *B. bovis* under natural conditions, and *B. bovis* is more pathogenic than *B. bigemina* [11]. In Colombia, a molecular study of cattle (*n* = 1432) revealed that the *Babesia*-positive rate is 31.6% (24.2% for *B. bigemina* and 14.4% for *B. bovis*) [7].

Bovine anaplasmosis is a major tick-borne bacterial disease in cattle [12]. The causative agents in the genus *Anaplasma* include *Anaplasma marginale*, *Anaplasma centrale*, *Anaplasma phagocytophilum*, and *Anaplasma bovis* [12,13]. *Anaplasma marginale* infects erythrocytes and is highly pathogenic in cattle with a wide distribution in tropical and subtropical regions [13,14]. In cattle aged > 2 years, *A. marginale* causes persistent fever, lethargy, icterus, weight loss, abortion, decreased milk yield, and death in more than 50% of untreated animals [14,15]. *Anaplasma centrale*, which is less pathogenic than *A. marginale*, causes mild symptoms in cattle [13]. *Anaplasma phagocytophilum* is an obligate intracellular bacterium that infects granulocytes and is distributed worldwide [13,15]. It is a zoonotic pathogen that causes tick-borne fever in ruminants and induces high fever, respiratory symptoms, leucopoenia, abortion, and sudden decreases in milk yield [13,15]. *Anaplasma bovis*, a monocytotropic species, has been detected in ruminants in many countries. Asymptomatic infection has been documented; however, it can cause fever, anaemia, weight loss, and occasional abortion and death [13]. *Anaplasma platys* usually infects dogs and is generally transmitted by brown dog ticks (*Rhipicephalus sanguineus*). However, in recent studies, strains genetically related to *A. platys* (*A. platys*-like) were detected in ruminants (sheep, goats, deer, camels, and cattle) [16,17,18,19]. For instance, a survey of beef cattle (*n* = 400) in the Brazilian Pantanal detected *A. platys*-like in 4.75% of the tested animals showing no anaemia or other clinical signs [20].

Cattle are of substantial economic importance for the livestock industry in Bolivia. The expansion of cattle ranching was expected since Bolivia began exporting beef to China in 2019 [21]. In Bolivia, only a few serological studies have investigated the prevalence of TBDs more than two decades ago [22,23]. Since then, detailed epidemiological reports including genetic data for pathogens circulating in the area are lacking. Therefore, this study aimed to detect and characterise tick-borne pathogens in different cattle breeds in Santa Cruz, Bolivia, using molecular methods.

## 2. Materials and Methods

### 2.1. Blood Sampling and DNA Extraction

**Figure 1:**
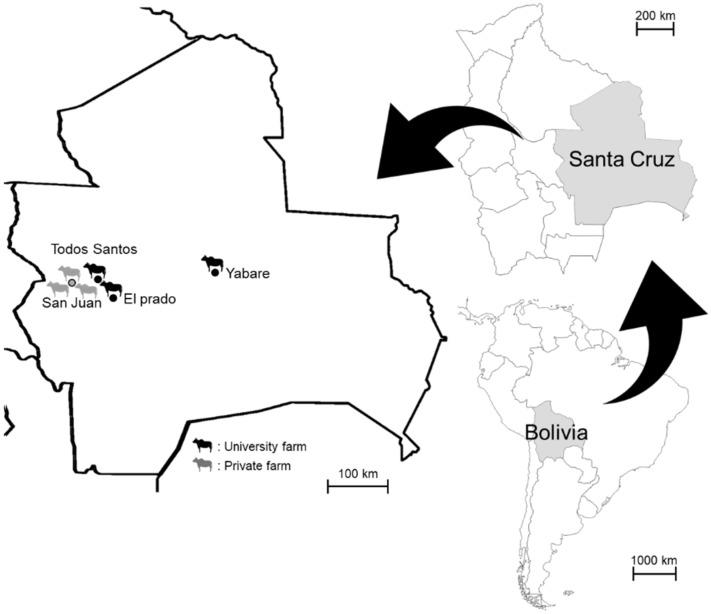
Geographical location of the study farms in Santa Cruz, Bolivia.

Blood samples were collected from pastured cattle (age of over 18 months, regardless of sex) at three farms managed by the Autonomous University Gabriel Rene Moreno (University Farm 1 at El Plado, University Farm 2 at Todos Santos, and University Farm 3 at Yabare) and three private farms located in San Juan, Santa Cruz, between December 2019 and March 2020 (Figure 1). A total of 104 individuals from the following 8 breeds were included: Nelore (*n* = 41), Holstein (*n* = 10), Gyr (*n* = 10), Gyrolando (*n* = 8), Mestizo (*n* = 2), Senepol (*n* = 10), Neloblanca (*n* = 1), and Criollo (*n* = 22). The sampled animals were randomly selected from each farm. Approximately 2–3 mL of cattle blood was collected from the jugular vein into EDTA tubes. DNA was extracted from 500 µL of blood using DNAzol (MRC, Cincinnati, OH, USA). All animal handling procedures were conducted in accordance with the guidelines established by the Animal Experiment Committee of the Graduate School of Veterinary Medicine, Hokkaido University (Sapporo, Japan). This study was approved by the Institutional Committee for the Care and Use of Experimental Animals at Autonomous University Gabriel Rene Moreno (CICUAE, 2015, No. 008/19).

### 2.2. Detection and Characterisation of Babesia spp.

*Babesia*, *Theileria*, and *Hepatozoon* were screened by nested BTH PCR using the primer sets BTH 1st F/BTH 1st R and BTH 2nd F/BTH 2nd R for the primary and secondary rounds, respectively (Table 1). This PCR amplified nearly the full length of the 18S ribosomal RNA gene (rDNA) (1400–1600 bp) [24]. The PCR was carried out in a 20.0 μL reaction mixture containing 10.0 μL of 2× Gflex PCR Buffer (Mg^2+^, dNTP plus), 400 nM of Tks Gflex™ DNA Polymerase (Takara Bio, Shiga, Japan), 400 nM of each primer, 1.0 μL of DNA template (or 10-fold diluted first PCR product), and sterilized water. The reaction was performed at 94 °C for 1 min, followed by 45 cycles at 98 °C for 10 s, 55 or 60 °C for 15 s, and 68 °C for 90 s, and a final step at 68 °C for 5 min. The PCR products were separated by electrophoresis on a 1.5% agarose gel stained with Gel-Red (Biotium, Hayward, CA, USA) and visualised under UV light (Supplementary Figure S1). Each assay included *Theileria parva* DNA detected in our previous study [25] and sterilised water as positive and negative controls, respectively.

### 2.3. Detection and Characterisation of Anaplasmataceae

*Anaplasmataceae* was screened by EHR PCR using the primers EHR16SD and EHR16SR (Table 1). This PCR amplified approximately 345 bp of the V1 hypervariable region of the 16S rDNA of *Anaplasmataceae* [26].

To further characterise *Anaplasmataceae* detected by EHR PCR, additional semi-nested PCRs targeting the heat shock protein (*groEL*) and citrate synthase (*gltA*) genes were employed [16]. A partial *gltA* gene sequence (approximately 630 bp) of *A. platys* and related strains was amplified in either of the two semi-nested PCRs. Initially, PCR was conducted using the primer sets Pglt-F/Pglt-R1 and Pglt-F/Pglt-R2 for the primary and secondary rounds of semi-nested PCR, respectively (Table 1). Alternatively, when the reaction was negative, another PCR was conducted using the primer sets Pglt-L-F1/Pglt-L-R and Pglt-L-F2/Pglt-L-R for the primary and secondary rounds, respectively (Table 1). Approximately 373 bp of the *groEL* gene sequence was amplified with either of the following primer combinations: Pgro-F-F/Pgro-F-R1 for the primary and Pgro-F-F/Pgro-F-R2 for the secondary round, Pgro-L-F1/Pgro-L-R for the primary and Pgro-L-F2/Pgro-L-R for the secondary round, or Pgro-F1/Pgro-R for the primary and Pgro-F2/Pgro-R for the secondary round (Table 1).

The PCR was conducted as described above with the annealing temperatures listed in Table 1. Tick DNA samples positive for *Anaplasma* in our previous study [27,28] and sterilised water were included in each PCR run as positive and negative controls, respectively. The PCR products were separated by electrophoresis on a 1.0% agarose gel stained with Gel-Red and visualised under UV light (Supplementary Figure S1).

### 2.4. Sanger Sequencing

All amplicons of the second *groEL* and second *gltA* PCRs for *Anaplasmataceae* and the second BHT PCR were purified using ExoSAP-IT (USB Corporation, Cleveland, OH, USA). The purified products were sequenced using the BigDye Terminator version 3.1 Cycle Sequencing Kit (Applied Biosystems, Foster City, CA, USA), and an ABI Prism 3130xl Genetic Analyzer (Applied Biosystems).

### 2.5. Data Analysis

Raw sequence data were edited by merging the forward and reverse sequences, followed by the removal of primer annealing sites using ATGC version 9.1 (GENETYX Corporation, Tokyo, Japan). Phylogenetic trees were constructed based on the partial sequences of *groEL* and *gltA* for *Anaplasma* and 18S rDNA for *Babesia*. The nucleotide sequences were aligned with representative sequences of known *Anaplasma* and *Babesia* species available in GenBank as implemented in MEGA7 [29]. Phylogenetic trees were constructed using the maximum likelihood (ML) method with 1000 bootstrap replicates. The best evolutionary models for the sequence data were determined based on the Akaike information criterion using MEGA7 [29]. The sequences obtained in this study were submitted to the DNA Data Bank of Japan (DDBJ) (18S rDNA sequences of *B. bigemina*, LC645216-LC645223; 18S rDNA sequence of *B. bovis*, LC645224; *gltA* gene sequences of *A. platys*-like, LC645225-LC645237; *gltA* gene sequence of *A. marginale*: LC645238; *groEL* gene sequence of *A. platys*-like, LC645239-LC645260).

## 3. Results

### 3.1. Detection and Characterisation of Babesia

**Figure 2:**
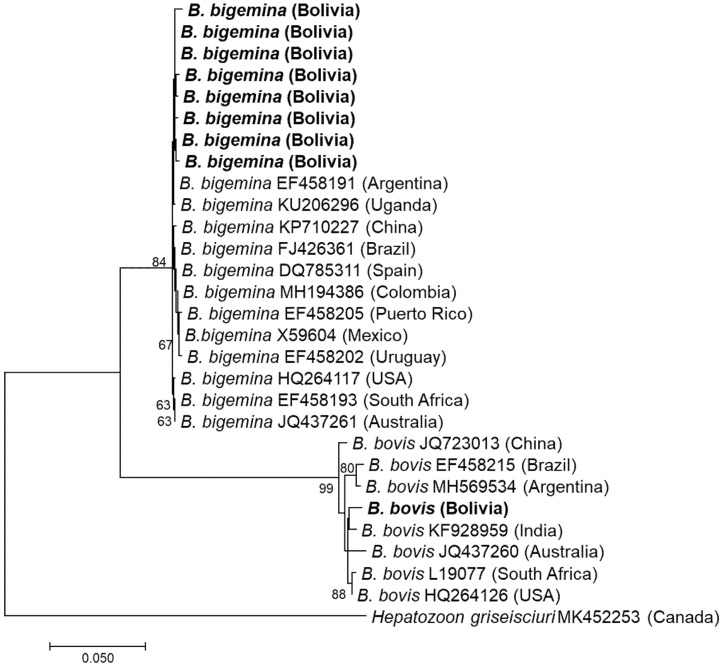
Phylogenetic tree based on 18S rDNA sequences of *Babesia* species. The phylogenetic tree was constructed using MEGA7 based on the maximum likelihood method using the Tamura 3-parameter model. Only bootstrap values greater than 60% are indicated. The sequences obtained in this study are shown in bold. The geographic origin (country) of each sequence/strain is provided in parentheses.

BTH PCR was positive in nine cattle: five at University Farm 1, three at University Farm 2, and one at University Farm 3. The cattle breeds that were positive for infection were Nelore (*n* = 2), Holstein (*n* = 5), Gyrolando (*n* = 1), and Criollo (*n* = 1). All amplicons were successfully sequenced using the Sanger method. The species detected were *B. bigemina* (*n* = 8) and *B. bovis* (*n* = 1) (Figure 2).

Eight 18S rDNA sequences of *B. bigemina* showed one and seven mismatched bases and showed the highest sequence identity (99.5–99.9%) with 18S rDNA of *B. bigemina* vaccine strain ‘S1A’ reported from Argentina (EF458191). One sequence of *B. bovis* had the highest identity (1387/1403, 98.9%) with 18S rDNA of a *B. bovis* strain from the blood of cattle in India (KF928959).

### 3.2. Detection and Characterisation of Anaplasmataceae

By EHR PCR detection, *Anaplasmataceae* infection was positive in 32 cattle: 8 from University Farm 1, 15 from University Farm 2, 7 from University Farm 3, 1 from Private Farm 2, and 1 from Private Farm 3. The infected cattle breeds were Nelore (*n* = 4), Holstein (*n* = 8), Gyr (*n* = 6), Gyrolando (*n* = 7), and Criollo (*n* = 7).

**Figure 3:**
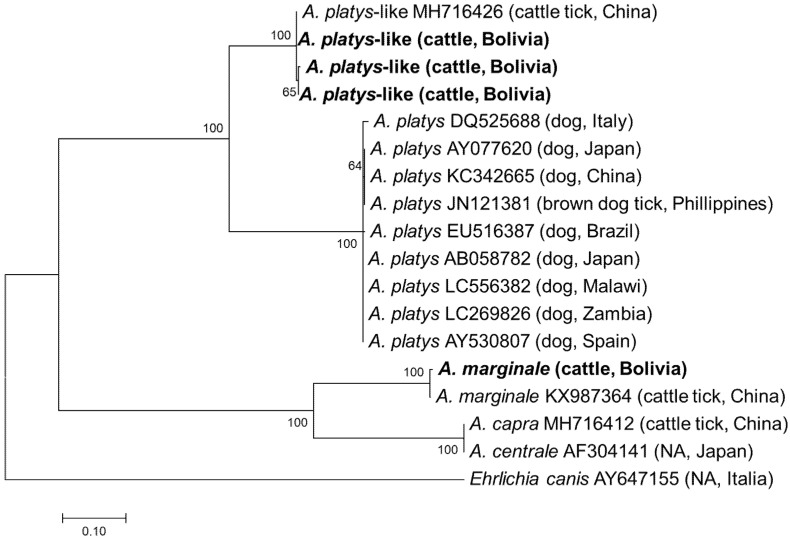
Phylogenetic tree based on *gltA* sequences in *Anaplasma* species. The phylogenetic tree was constructed using MEGA7 based on the maximum likelihood method using the Kimura 2-parameter model. Only bootstrap values greater than 60% are indicated. The sequences obtained in this study are shown in bold. The host animal and geographic origin (country) of the sequence/strain are provided in parentheses. NA stands for not available.

**Figure 4:**
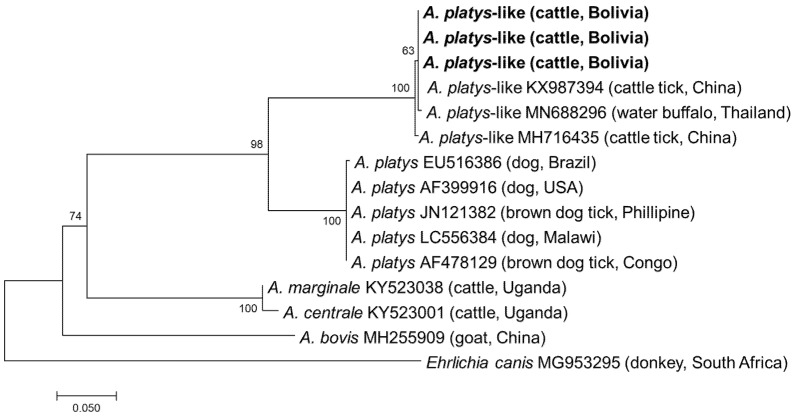
Phylogenetic tree based on the *groEL* sequences of *Anaplasma* species. The phylogenetic tree was constructed using MEGA7 based on the maximum likelihood method, using the Tamura 3-parameter model. Only bootstrap values greater than 60% are indicated. The sequences obtained in this study are shown in bold. The geographic origin (country) of each sequence/strain is provided in parentheses.

All samples positive by EHR PCR were subjected to semi-nested PCRs targeting the *gltA* and *groEL* genes. Although PCR amplicons were obtained from all 32 samples, *gltA* and *groEL* were only successfully sequenced in 14 and 22 samples, respectively (Supplementary Table S1). In the remaining samples, sequencing failed due to mixed signals. Finally, a sequencing analysis of the purified amplicons identified four and three different *gltA* and *groEL* sequences, respectively. Three *gltA* sequences obtained from thirteen samples showed the highest sequence identity (628–630/630, 99.7–100.0%) with the *A. platys*-like strain reported from *R. microplus* in China (MH716426), while one *gltA* sequence had the highest identity (611/633, 96.5%) with *A. marginale* reported from *R. microplus* in China (KX987364). The three *groEL* sequences obtained had 1–5 nucleotide mismatches with each other and showed the highest identity (370–372/373, 99.2–99.7%) with an *A. platys*-like strain reported from *R. microplus* in China (MH716435). Phylogenetic trees based on *gltA* and *groEL* are shown in Figure 3 and Figure 4, respectively. The *gltA* sequences of *A. platys* were divided into two clades in both trees. The clade including *A. platys*-like detected in this study was composed of *A. platys*-like detected from cattle ticks (*R. microplus*), cattle, camels, and water buffalo, while the other clade consisted of *A. platys* reported from dogs and brown dog ticks (*R. sanguineus*) (Figure 3 and Figure 4).

### 3.3. Co-Infection

Seven of nine cattle infected with *B. bigemina* were also infected with *A. platys*-like (Supplementary Table S2). These included four animals at University Farm 1 and three animals at University Farm 2. One *B. bigemina*-infected animal at University Farm 1 was co-infected with *A. marginale* (Supplementary Table S1). The cattle breeds that were positive for co-infection were Nelore (*n* = 2), Holstein (*n* = 5), and Gyrolando (*n* = 1) (Table 2, Supplementary Table S2).

## 4. Discussion

Babesiosis and anaplasmosis are among the most important TBDs in cattle. Although there is increasing evidence for their high prevalence worldwide, few studies have focused on resource-limited countries, such as Bolivia. The lack of epidemiological data for the occurrence of TBDs not only leads to misdiagnosis and treatment delays but also hinders the design of proper tick control measures.

Bovine babesiosis is a worldwide TBD caused by *B. bovis* and *B. bigemina* in Latin America [7]. This study is the first molecular survey of TBDs in cattle in Bolivia and confirmed the presence of both *B. bovis* and *B. bigemina*. Most *Babesia* detected in this study were *B. bigemina*, which is pathogenic and thus poses a significant challenge to livestock health. These results are in contrast to those of previous serological studies showing that the seroprevalence rate of *B. bovis* is higher than that of *B. bigemina* in two different surveyed locations, Bolivian Chaco and Santa Cruz [22,23]. However, molecular studies in other Latin American countries, including Argentina, Brazil, and Colombia, have also reported a higher molecular prevalence of *B. bigemina* than *B. bovis* [8,30,31]. The presence of two pathogenic *Babesia* species indicates poor tick management procedures in the surveyed farms. Although diminazene aceturate and imidocarb dipropionate have been used to treat animal babesiosis, several studies have suggested the possible development of diminazene aceturate resistance in *Babesia* parasites [32,33]. Therefore, proper tick control programs as well as the appropriate use of chemotherapeutic agents are necessary.

*Anaplasma* infection in cattle is mainly caused by *A. marginale*, *A. centrale*, *A. phagocytophilum*, and *A. bovis*. Other *Anaplasma* species of unknown pathogenicity, such as *A. platys*-like and *Anaplasma capra*, have also been detected in cattle [16,19,34,35]. In the present study, *A. marginale*, a pathogenic species, was detected. In addition, *A. platys*-like infection in cattle in Bolivia was first reported in this study. Although *A. platys* is primarily infectious and pathogenic to dogs, there is also evidence that *A. platys*-like infects ruminants, including cattle in Algeria, Brazil, and Egypt, goats in Tunisia, camels in Egypt, water buffalo in Thailand, sheep in Tunisia, and red deer in China [18,19,20,26,36,37,38,39]. The sequences of *A. platys*-like detected in this study clustered with those detected in *R. microplus* in China, cattle in Egypt, and water buffalo in Thailand, and formed a distinct cluster from those reported in dogs and brown dog ticks (Figure 3 and Figure 4). These results support the geographically widespread distribution of *A. platys*-like infection in ruminants. *Anaplasma platys*-like strains were found to infect ruminant neutrophils instead of platelets [20]. Although the pathogenicity of these *A. platys*-like strains is unknown, a study from Brazil reported that the cattle infected with *A. platys*-like did not show any clinical signs, including anaemia [20]. Nonetheless, further studies are needed to investigate their interaction with host ticks and other microorganisms, including pathogenic *Anaplasma*.

This study employed cattle reared on farms with different tick control measures. In private farms, external pour-on antiparasitics (ACIENDEL PLUS, Biogénesis Bagó, Casa Matriz, Argentina) and injection (Dectomax^®^ Injectable solution, Zoetis, NJ, USA) were used for tick prevention twice a year (March and September). In contrast, in the university farms, acaricide (ECTOSULES 6% SPILLED, Laboratorio Microsules, Uruguay) was used for tick prevention twice a year (February and August). In recent years, acaricide resistance in ticks has been a remarkable problem worldwide, and resistance to almost all chemicals has been observed in *R. microplus* [40,41]. The difference in tick control measures may explain the higher infection rate of TBDs in university farms than in private farms. Additionally, the cattle breeds differed among farms. Of note, University Farm 1, where the Holsteins were kept, had the highest infection rate (Table 2). Several studies have shown that genetic factors are associated with resistance to tick infestations and babesiosis, with *Bos taurus indicus* (e.g., Nelore) being more resistant than *Bos taurus taurus* (e.g., Holstein) [42,43]. In fact, *B. taurus indicus* is less likely to be infected with tick-borne pathogens because it shows tick resistance [44]. In Brazil, Angus cattle show a significantly higher rate of *Babesia* infection than that of Nelore cattle [42,44]. Another possible explanation for the high infection rate in Holstein cattle is poor adaptation to the climate in Bolivia. Holstein is a cold-hardy European breed that may be less resistant to the tropical climate of South America. In fact, although the health status of cattle was not evaluated in the present study, Holstein cattle showed a higher frequency of clinical signs, such as loss of appetite and decrease in body weight, than that of Nelore cattle (data not shown). In Bolivia, a variety of hybrids are bred locally, and these may be highly adaptable to the Bolivian environment.

In this study, most cattle infected with *Babesia* were infected with *A. platys*-like. Co-infection with *Babesia* and *Anaplasma* is a common finding in cattle: *A. marginale* and *B. bigemina*, and *Anaplasma* sp. and *B. bigemina* have been simultaneously detected in the same individuals in Egypt and Ethiopia, respectively [45,46]. The effect of mixed infections on clinical outcomes needs to be evaluated in the future. 

In the past two serological studies conducted in Bolivia, three tick-borne pathogens, namely *B. bovis*, *B. bigemina*, and *A. marginale*, were detected from cattle, and relatively high seroprevalence was observed: *B. bovis* (66.1% and 64.2%), *B. bigemina* (32.1% and 46.3%), and *A. marginale* (20.5% and 38.8%) [22,23]. Though this study also detected the same pathogens, overall infection rates were lower than those observed in the previous serological studies. PCR-based assays detect pathogens’ DNA, and the results depend on the presence of the target DNA in the tested blood. In contrast, serological assays detect the antibodies resulting from immune response to the infection and thus do not show infection status at the time of sampling. Although this difference hinders a direct comparison between studies, the results correctively indicate that these pathogens have been endemic for at least decades in Bolivia. There is a need to conduct a longitudinal survey to monitor the prevalence of TBDs in Bolivia.

One of the biggest limitations of this study is that blood samples were collected from a limited number of cattle. The variation in sample size among cattle breeds makes it difficult to conduct a statistical analysis of the effect of breed on TBDs. Additionally, the occurrence of TBDs is attributed to interactions between arthropod vectors and hosts, and these interactions are influenced by climatic factors, such as temperature, relative humidity, and precipitation [47]. Moreover, the samples were only collected from geographically limited areas, which may have masked other TBDs prevalent in Bolivia. Thus, this cross-sectional survey may have underestimated the TBD status in cattle. Further studies employing larger sample sizes from geographically diverse locations are needed to evaluate the overall significance of TBDs in the cattle industry in Bolivia. It is also of great importance to conduct a survey on ticks, including *R. microplus,* which has been observed on the body surface of the cattle examined in this study (data not shown), since the presence of *Babesia* and *Anaplasma* has been detected from this tick species in other countries [11,12,14].

## 5. Conclusions

This is the first report to provide molecular evidence for four tick-borne pathogens, namely *B. bovis*, *B. bigemina*, *A. marginale*, and *A. platys*-like, in cattle in Bolivia. The results of this study provide information about the prevalence of TBDs as well as updated molecular data for the studied areas. This information is important for understanding the epidemiology of TBDs and interactions among pathogens and is expected to help in formulating appropriate and improved control strategies for pathogens in the area, consequently reducing losses in the cattle industry. Since *A. platys*-like infection in cattle is insufficiently understood, further efforts are needed to clarify their pathogenicity in animals and their interaction with other pathogens in ticks. Longitudinal sampling of cattle from wider geographic origins is needed to obtain a comprehensive view of the prevalence and epidemiological consequences of TBDs in Bolivia.

## Figures and Tables

**Table 1:** Primers used in this study.

Primer	Sequence (5′-3′)	Target Gene	Organism	Annealing Temperature (°C)	Purpose	Reference
EHR16SD	GGTACCYACAGAAGAAGTCC	16S rDNA	*Anaplasmataceae*	55	PCR	[26]
EHR16SR	TAGCACTCATCGTTTACAGC					
Pglt-F	ATGAWAGAAAAWGCTGTTTT	*gltA*	*Anaplasma*	60	Sequencing	[16]
Pglt-R1	TCATGRTCTGCATGCATKATG					
Pglt-R2	CATGCATKATGAARATMGCAT					
Pglt-L-F1	GATGCWCATCCYATSGCMATGT	*gltA*	*Anaplasma*	60	Sequencing	[16]
Pglt-L-F2	CGTGMTSGCTATAGCGMAART					
Pglt-L-R	TCAYACCATTGDGAYRCCCAT					
Pgro-F1	TTGATCATCGCTGAAGACGT	*groEL*	*Anaplasma*	60	Sequencing	[16]
Pgro-F2	ACTCTCGTCTTGAACAAGCT					
Pgro-R	CCACTCTGTCTTTACGCTCT					
Pgro-F-F	AAATGKCAAATACGGTWGTC	*groEL*	*Anaplasma*	60	Sequencing	[16]
Pgro-F-R1	ACAACACCTTCCTCKACAGC					
Pgro-F-R2	CTGKCTTTRCGYTCTTTAACTTC					
Pgro-L-F1	GAYGGTATGCAGTTTGATCGCG	*groEL*	*Anaplasma*	60	Sequencing	[16]
Pgro-L-F2	ATGCAGTTTGATCGCGGWTATC					
Pgro-L-R	CAGCRAGGTCGAAYGCAATAC					
BTH 1st F	GTGAAACTGCGAATGGCTCATTAC	18S rDNA	*Babesia*	55	PCR	[24]
BTH 1st R	AAGTGATAAGGTTCACAAAACTTCCC		*Theileria*			
BTH 2nd F	GGCTCATTACAACAGTTATAGTTTATTTG		*Hepatozoon*			
BTH 2nd R	CGGTCCGAATAATTCACCGGAT					
BTH18SinterF	ATTTTCCGACTCCTTCAGCA	18S rDNA	*Babesia*	NA	Sequencing	This study
BTH18SinterR	AACTAAGAACGGCCATGCAC					

Note: NA, not applicable.

**Table 2:** Infection rate of *Babesia* and *Anaplasma*.

Region	Breed	Number	*Babesia*	*Anaplasma* (EHR PCR)	*Anaplasma* (*groEL*)	*Anaplasma* (*gltA*)	Co-Infection
University Farm 1	Holstein	10	5 (50%)	8 (80%)	8 (80%)	5 (50%)	5 (50%)
University Farm 2	Nelore	10	2 (20%)	2 (20%)	1 (10%)	-	2 (20%)
	Gyr	10	-	6 (60%)	6 (60%)	3 (30%)	-
	Gyrolando	8	1 (13%)	7 (88%)	5 (63%)	1 (13%)	1 (13%)
University Farm 3	Criollo	22	1 (5%)	7 (32%)	2 (9%)	5 (23%)	-
Private Farm 1	Nelore	10	-	-	-	-	-
	Mestizo	2	-	-	-	-	-
	Senepol	10	-	-	-	-	-
Private Farm 2	Nelore	11	-	1 (9%)	-	-	-
	Neloblanca	1	-	-	-	-	-
Private Farm 3	Nelore	10	-	1 (10%)	-	-	-

Note: -, not amplified.

## Data Availability

*Babesia* and *Anaplasma* sequences obtained in this study were submitted to the DNA Data Bank of Japan (DDBJ) under the following accession numbers (18S rDNA sequences of *B. bigemina*, LC645216-LC645223; 18S rDNA sequence of *B. bovis*, LC645224; *gltA* gene sequences of *A**. platys*-like, LC645225-LC645237; *gltA* gene sequence of *A. marginale*: LC645238; *groEL* gene sequence of *A**. platys*-like, LC645239-LC645260).

## Supplementary Materials

The following are available online at https://www.mdpi.com/article/10.3390/vetsci8090188/s1, Table S1: Results of PCR and Sanger sequencing of *Anaplasmataceae*-positive samples. Table S2: List of samples co-infected with *Babesia* and *Anaplasma*. Figure S1: Agarose gel electrophoresis of (a) BTH PCR products, (b) EHR PCR products, (c) *gltA* PCR (primer set 1) products, and (d) *groEL* PCR (primer set 1) products.
