# Supplementary material for: Molecular Survey of Babesia and Anaplasma Infection in Cattle in Bolivia
Source: Vet Sci. 2021 Sep 7;8(9):188. doi: 10.3390/vetsci8090188 (PMC8473379; doi:10.3390/vetsci8090188)
Supplement: Supplementary file 1 [file vetsci-08-00188-s001.zip › vetsci-1341328-supplementary.pdf]

Supplementary Data

Table S1. Results of PCR and Sanger sequencing of *Anaplasmataceae*-positive samples.

Cattle ID	EHR PCR	*gltA* PCR (primer set 1)	*gltA* PCR (primer set 2)	*groEL* PCR (primer set 1)	*groEL* PCR (primer set 2)	*groEL* PCR (primer set 3)
U1-H5	+	-	M	*A. platys*-like	M	-
U1-H517	+	M	*A. platys*-like	*A. platys*-like	*A. platys*-like	M
U1-H520	+	*A. platys*-like	-	*A. platys*-like	M	M
U1-H527	+	M	M	*A. platys*-like	M	M
U1-H530	+	*A. platys*-like	*A. platys*-like	*A. platys*-like	*A. platys*-like	*A. platys*-like
U1-H536	+	M	M	*A. platys*-like	-	*A. platys*-like
U1-H1210	+	*A. platys*-like	M	*A. platys*-like	-	*A. platys*-like
U1-H1212	+	*A. platys*-like	M	*A. platys*-like	-	*A. platys*-like
P2-N275	+	-	-	M	M	M
P3-N8	+	M	M	M	M	M
U2-G332	+	M	-	*A. platys*-like	M	*A. platys*-like
U2-G347	+	M	M	*A. platys*-like	M	M
U2-G376	+	M	M	*A. platys*-like	M	M
U2-G431	+	*A. platys*-like	*A. platys*-like	*A. platys*-like	*A. platys*-like	*A. platys*-like
U2-G516	+	*A. platys*-like	*A. platys*-like	*A. platys*-like	*A. platys*-like	M
U2-G521	+	*A. platys*-like	*A. platys*-like	*A. platys*-like	M	M
U2-G1114	+	M	M	*A. platys*-like	M	*A. platys*-like
U2-G1154	+	M	-	*A. platys*-like	M	M

U2-G1170	+	M	-	*A. platys*-like	M	M
U2-G2136	+	M	-	*A. platys*-like	M	M
U2-G3862	+	M	-	*A. platys*-like	M	M
U2-G4172	+	M	M	M	M	-
U2-G5250	+	*A. marginale*	M	M	M	-
U2-N1068	+	M	M	*A. platys*-like	-	M
U2-N1108	+	M	-	M	-	M
U3-C5102	+	M	*A. platys*-like	*A. platys*-like	-	-
U3-C2086	+	*A. platys*-like	*A. platys*-like	*A. platys*-like	-	-
U3-C1220	+	*A. platys*-like	*A. platys*-like	M	-	M
U3-C5016	+	*A. platys*-like	*A. platys*-like	M	-	M
U3-C9210	+	M	M	M	-	M
U3-C4078	+	*A. platys*-like	*A. platys*-like	M	M	M
U3-C4250	+	-	M	M	M	M

gltA PCR primer set 1, Pglt-F, Pglt-R1, and Pglt-R2. *gltA* PCR primer set 2, Pglt-L-F1, Pglt-L-F2, and Pglt-L-R. *groEL* PCR primer set 1, Pgro-F1, Pgro-F2, and Pgro-R. *groEL* PCR primer set 2, Pgro-F-F, Pgro-F-R1, and Pgro-F-R2. *groEL* PCR primer set 3, Pgro-L-F1, Pgro-L-F2, and Pgro-L-R.

+, PCR positive; -, PCR negative; M, mixed signals in Sanger sequencing data.

Table S2. List of samples co-infected with *Babesia* and *Anaplasma*.

Cattle ID	Farm	***Babesia* species**	***Anaplasma* species**
U1-H5	University Farm 1	*B. bigemina*	*A. platys*-like
U1-H517	University Farm 1	*B. bigemina*	*A. platys*-like
U1-H536	University Farm 1	*B. bigemina*	*A. platys*-like
U1-H1210	University Farm 1	*B. bigemina*	*A. platys*-like
U1-H1212	University Farm 1	*B. bigemina*	*A. platys*-like
U2-G5250	University Farm 2	*B. bigemina*	*A. marginale*
U2-N1068	University Farm 2	*B. bigemina*	*A. platys*-like
U2-N1108	University Farm 2	*B. bigemina*	*A. platys*-like

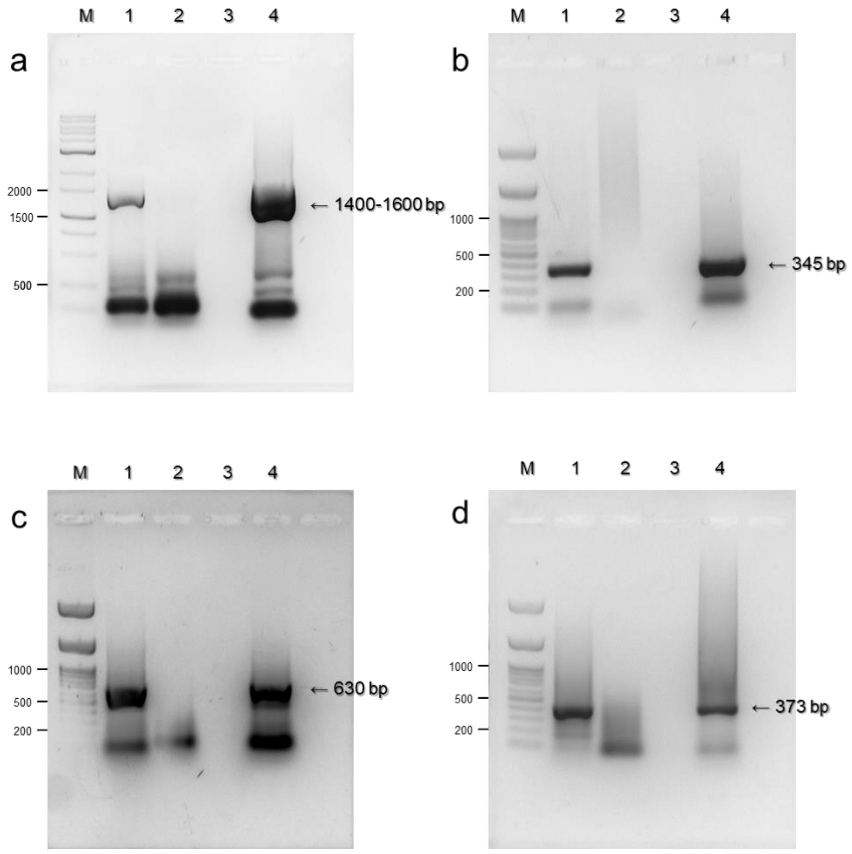

Figure S1. Agarose gel electrophoresis of (a) BTH PCR products, (b) EHR PCR products, (c) *gltA* PCR (primer set 1) products, and (d) *groEL* PCR (primer set 1) products. The expected amplified product size is shown with an allow. Lanes M, 1, 2, 3, and 4 indicate DNA size marker, PCR-positive sample, PCR-negative sample, negative control (water), and positive control (*Babesia* or *Anaplasma* DNA), respectively.
